# Testing Measurement Invariance Across Gender in a Low Resource Setting Using the Computerized Battery for Neuropsychological Evaluation of Children (BENCI)

**DOI:** 10.1002/alz.094280

**Published:** 2025-01-09

**Authors:** Rachel W Maina, Jelte Wicherts

**Affiliations:** ^1^ Aga Khan University, Nairobi Kenya; ^2^ Tilburg University, Tilburg Netherlands

## Abstract

**Background:**

Developing culture‐fair tests that measure constructs equivalently across different ethno‐lingual groups is challenging, given the diverse cultural variations that impact neurocognitive measurement. Multi‐level measurement invariance must be established before interpreting scores similarly across groups, both within and between cultures for meaningful comparisons.

**Method:**

We set out to test whether a neurocognitive tool (BENCI) behaves the same way across the males (n = 311) and the females group (n = 291) using measurement invariance testing with multigroup confirmatory factor analysis. We used a four factor model that had an excellent fit in a Kenyan pooled sample when modified to have four correlated first‐order factors i.e., Fluency, Reasoning, Memory, and Flexibility. We evaluated configural, metric, scalar and lastly strict invariance model and then tested whether the different restraint in the models resulted in a significant chi‐square difference.

**Result:**

The four‐factor model showed good model fit (RMSEA = .034, CFI = .959, TLI = .930, χ2 (92, n = 604) = 154.309, p < .001), with metric measurement invariance (RMSEA = .033, CFI = .957, TLI = .933, χ2 (100, n = 604) = 164.368, p < .001; Δχ2 = 10.059, DF = 8, p = 261, ΔCFI = 0.002), scalar measurement invariance (RMSEA = .032, CFI = .957, TLI = .938, χ2 (108, n = 604) = 172.831, p < .001; Δχ2 = 8.463, p = .390, ΔCFI = 0.000), and the strict measurement invariance (RMSEA = .031, CFI = .955, TLI = .940, χ2 (118, n = 604) = 186.563, p < .001; Δchi‐square = 22.195, df = 18, p = .223, ΔCFI 0.002) between the females and males.

**Conclusion:**

Metric, scalar and strict invariance is achieved indicating that indicator items, indicator intercepts (same starting point on the BENCI) and residuals (except Visual Memory Delayed CA residuals) of the BENCI are comparable across the male and female samples, and we can compare the association of BENCI with other invariant constructs across the two groups, including the latent mean comparisons. These results are promising and suggest that cross‐gender comparisons may also be tenable for other clinical group comparisons in Kenya and cross‐cultural data harmonization in multi‐national research programs.

